# Phylogenomic Analysis of Metagenome-Assembled Genomes Deciphered Novel Acetogenic Nitrogen-Fixing *Bathyarchaeota* from Hot Spring Sediments

**DOI:** 10.1128/spectrum.00352-22

**Published:** 2022-06-01

**Authors:** Sushanta Deb, Subrata K. Das

**Affiliations:** a Department of Biotechnology, Institute of Life Sciences, Bhubaneswar, Odisha, India; University of Texas at San Antonio

**Keywords:** hot springs, metagenome-assembled genome, phylogeny, *Bathyarchaeota*, metabolic potential

## Abstract

This study describes the phylogenomic analysis and metabolic insights of metagenome-assembled genomes (MAGs) retrieved from hot spring sediment samples. The metagenome-assembled sequences recovered three near-complete genomes belonging to the archaeal phylum. Analysis of genome-wide core genes and 16S rRNA-based phylogeny placed the ILS200 and ILS300 genomes within the uncultivated and largely understudied bathyarchaeal phylum, whereas ILS100 represented the phylum *Thaumarchaeota*. The average nucleotide identity (ANI) of the bin ILS100 was 76% with Nitrososphaeria_archaeon_isolate_SpSt-1069. However, the bins ILS200 and ILS300 showed ANI values of 75% and 70% with Candidatus_Bathyarchaeota_archaeon_isolate_DRTY-6_2_bin_115 and Candidatus_Bathyarchaeota_archaeon_BA1_ba1_01, respectively. The genomic potential of *Bathyarchaeota* bins ILS200 and ILS300 showed genes necessary for the Wood-Ljungdahl pathway, and the gene encoding the methyl coenzyme M reductase (*mcr*) complex essential for methanogenesis was absent. The metabolic potential of the assembled genomes included genes involved in nitrogen assimilation, including nitrogenase and the genes necessary for the urea cycle. The presence of these genes suggested the metabolic potential of *Bathyarchaeota* to fix nitrogen under extreme environments. In addition, the ILS200 and ILS300 genomes carried genes involved in the tricarboxylic acid (TCA) cycle, glycolysis, and degradation of organic carbons. Finally, we conclude that the reconstructed *Bathyarchaeota* bins are autotrophic acetogens and organo-heterotrophs.

**IMPORTANCE** We describe the *Bathyarchaeota* bins that are likely to be acetogens with a wide range of metabolic potential. These bins did not exhibit methanogenic machinery, suggesting methane production may not occur by all subgroup lineages of *Bathyarchaeota*. Phylogenetic analysis support that both ILS200 and ILS300 belonged to the *Bathyarchaeota*. The discovery of new bathyarchaeotal MAGs provides additional knowledge for understanding global carbon and nitrogen metabolism under extreme conditions.

## INTRODUCTION

Despite being a significant and essential part of the microbial ecosystem in almost all environments, resources for archaeal research are limited (1). Several studies illustrated the abundance of archaea in the environmental samples (2–4). With the advent of next-generation sequencing and metagenomics approaches, a diverse group of novel *Candidatus* organisms of the domain *Archaea* and their genomes has been reconstructed and assembled. Moreover, the 16S rRNA gene sequence-based phylogenetic analysis is an essential tool for understanding the archaeal population dynamics in environmental samples (5). The archaeal research has successfully contributed a few novel archaeal genomes primarily to integrate the genomic information of a single organism (6, 7). Presently, the phyla *Thaumarchaeota*, *Aigarchaeota*, *Crenarchaeota*, *Korarchaeota*, and *Bathyarchaeota* have been proposed to constitute a superphylum, referred to as TACK (8, 9).

Several studies have demonstrated the distribution of *Thaumarchaeota* in marine and terrestrial environments and their importance in nitrification and carbon fixation (10). Members of the *Thaumarchaeota* are mostly uncultivated. As a result, metagenome-assembled genomes (MAG) provide an opportunity for understanding their metabolic adaptation in the process of evolution and niche expansion. Recent studies demonstrated that the assembled genome of *Thaumarchaeota* from marine water is distantly related to its affiliated members isolated from thermal habitats (11). In contrast, members of *Bathyarchaeota* are mostly reported from hot springs. These archaea are widespread in anoxic sediments and appeared as one of the most dominant phyla (12). Furthermore, genomic evidence suggested that members of the phylum *Bathyarchaeota* are involved in methane metabolism, a property found only in the phylum *Euryarchaeota* (6). In addition, acetogenesis, primarily restricted to the domain *Bacteria*, was also found in some lineages of *Bathyarchaeota* (13).

The energy source in the metabolic process suggests that hydrogen (H_2_) is the first electron donor leading to ATP synthesis in microbial cells by the enzyme hydrogenase. Till date, hydrogenase enzymes are found in the genomes of aquatic and terrestrial organisms and play a crucial role in carbon fixation (14). Acetyl-CoA produced during this process is essential for archaea’s acetogenesis, methanogenesis, and carbon fixation (12, 15). Apart from this, other electron donors such as NAD(P)H and ferredoxin have been reported in the energy-yielding process in hyperthermophilic methanogenic archaea (16). Furthermore, heterodisulfide reductase (Hdr-F_420_), an electron bifurcating complex that acts as an electron donor, is crucial for energy metabolism in methanogenic archaea. It is also essential to cycling coenzyme M and coenzyme B (CoM-CoB) associated with methanogenesis (17, 18).

Archaeal studies of the tropical hot springs located in the Indian subcontinent have received little attention. However, cultivation-based studies have shown the identification of several new species of bacteria from these hot springs (19). This study describes three metagenome-assembled genomes (MAGs) and identifies their phylogenetic affiliation. In addition, we report the metabolic potential of *Bathyarchaeota* bins.

## RESULTS AND DISCUSSION

### Genome characteristics.

The shotgun sequencing of the metagenome generated 50,483,993, 48,110,695, and 41,417,706 high-quality Illumina sequence reads for sample-1 (Surajkund, main source), sample-2 (Surajkund, surrounding area), and sample-3 (Bakreshwar), respectively. At the time of assembly of data, 46,004 contigs from Surajkund (main source), 22,119 contigs from Surajkund (surrounding area), and 23,777 contigs from Bakreshwar were available for analysis. *De novo* assembly and binning by tetranucleotide signatures identified *Bathyarchaeota* bins (ILS200 and ILS300) and a *Thaumarchaeota* bin (ILS100). The assembled genomes ILS100 and ILS200 were obtained from the metagenome of sample-2, whereas ILS300 was from sample-3. ILS100 represents a “*Candidatus* Thaumarchaeota” genome (2.22 Mbp) estimated to be ~98.06% complete. However, “*Ca.* Bathyarchaeota” genomes ILS200 (2.35 Mb) and ILS300 (1.75 Mb) were estimated to be ~98.88% and ~98.13% complete, as determined by the presence of single-copy marker genes (Table 1).

**TABLE 1:** Statistics for reconstructed archaeal genomes

Genomic characteristic	Data for MAGs		
Bin identity	“*Ca.* Thaumarchaeota” (ILS100)	“*‘Ca.* Bathyarchaeota” (ILS200)	“*Ca.* Bathyarchaeota” ILS300
BioSample ID	SAMN13381922	SAMN13565975	SAMN13381783
GenBank accession no.	WUQR00000000	WUQU00000000	WUQV00000000
Genome size (bp)	2,112,757	2,351,990	1,754,230
Completeness (%)	98.06	98.88	98.13
Contamination (%)	1.34	3.19	2.18
*N*_50_ (bp)	1,262,376	8,235	8,586
GC content (%)	52.29	42.24	47.44
tRNA genes	35	35	20
rRNA genes	3	4	4
Protein-coding genes	2,213	2,611	2,012
Hypothetical proteins	897	1,106	828
Genes annotated by COG^*a*^	1,516	1,982	1,360

a COG, clusters of orthologous genes.

### ANI and phylogenetic analysis.

**FIG 1:**
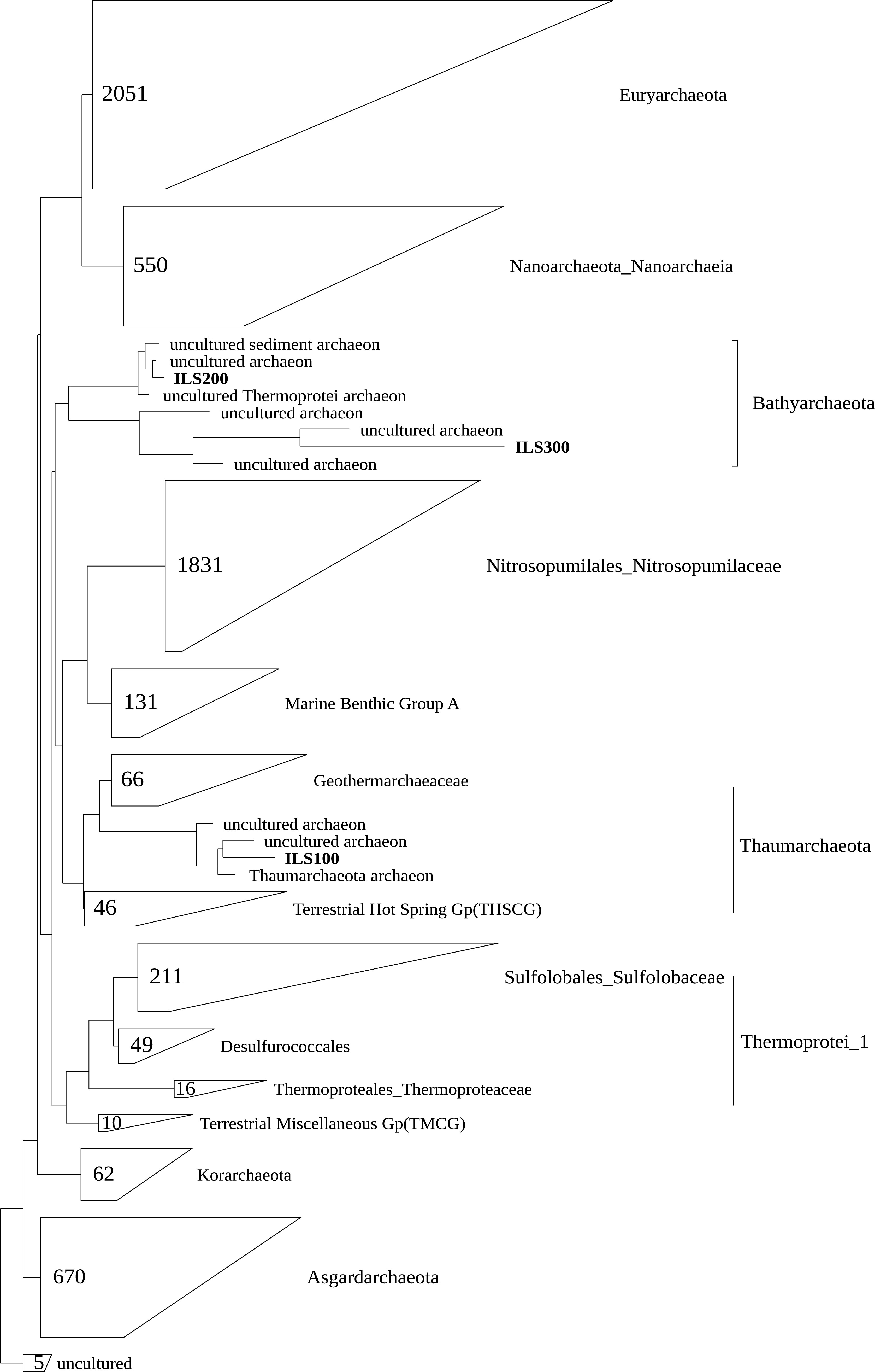
Maximum-likelihood phylogenetic tree computed using MAG-derived 16S rRNA gene sequences with the reference sequences from the database.

**FIG 2:**
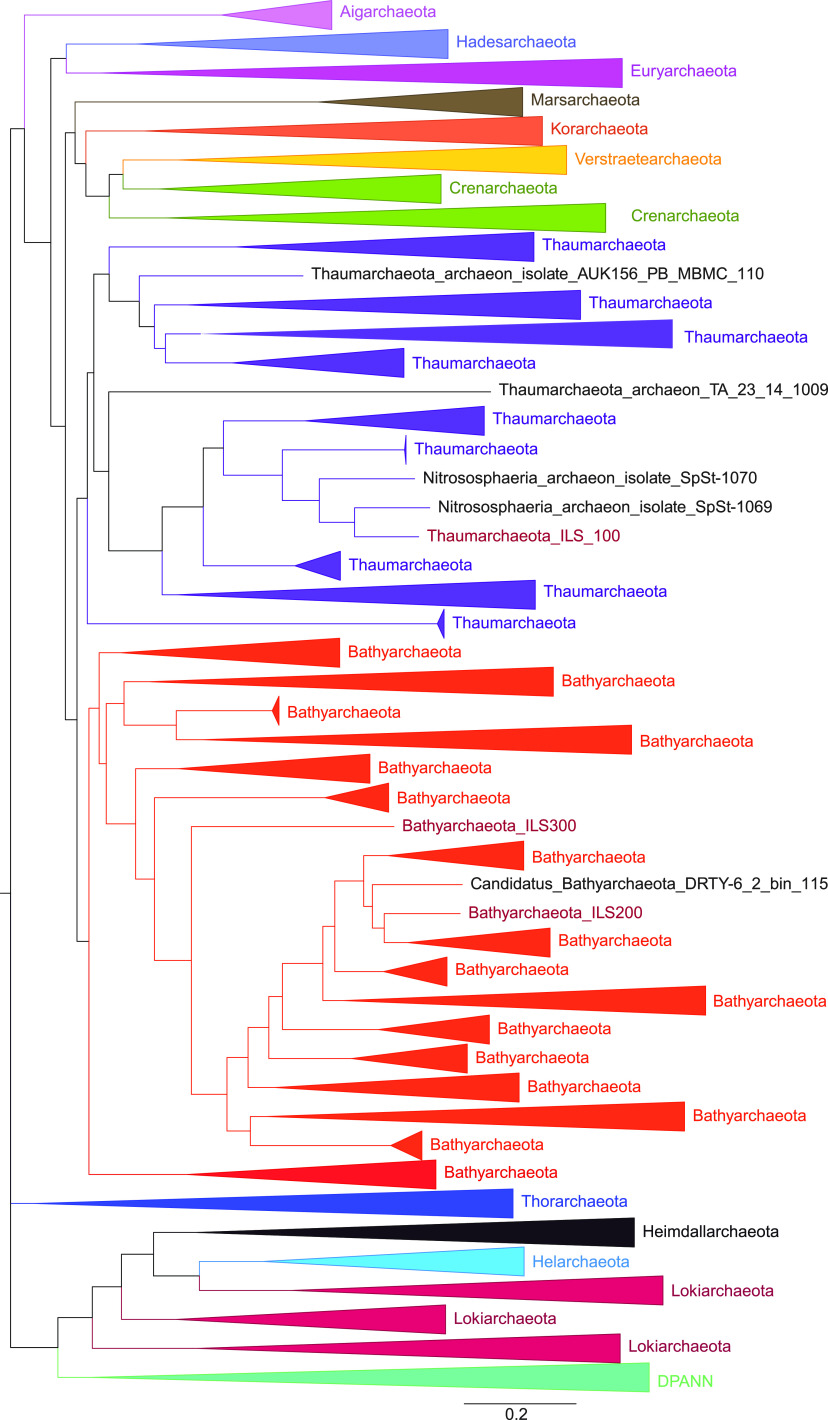
Phylogeny of reconstructed MAGs with respective archaeal clades. Maximum-likelihood tree of 1,265 archaea with concatenated amino acid sequences of 77 conserved single-copy marker proteins. The scale bar represents amino acid substitutions per sequence position.

The genome of ILS100 showed an ANI of 76% with Nitrososphaeria_archaeon_isolate_SpSt-1069 (7) (see Fig. S1 at https://figshare.com/s/d8c03fb25988b07c9479). Similarly, the genomes of ILS200 and ILS300 revealed 75% and 70% ANI with Candidatus_Bathyarchaeota_archaeon_isolate_DRTY-6_2_bin_115 and Candidatus_ Bathyarchaeota_archaeon_BA1_ba1_01, respectively (6) (Fig. S2 at the URL mentioned above). The ANI value of all three assembled genomes was less than 90%, so the similarity was at the level of different genera or even families. The phylogenetic affiliations of the assembled genomes of ILS100, ILS200, and ILS300 were compared with the those of reference genomes considering the 16S rRNA gene sequences and core genes. In the 16S rRNA phylogenetic tree, ILS100 clustered with the uncultured and largely understudied marine thaumarchaea. In comparison, ILS200 and ILS300 clustered with the uncultured archaeon of *Bathyarchaeota* (Fig. 1). Moreover, in phylogenetic tree based on core genes, ILS100 clustered within the phylum *Thaumarchaeota* lineage, while ILS200 and ILS300 clustered with the *Bathyarchaeota* phylum (Fig. 2). These results suggested that ILS100 belongs to the phylum *Thaumarchaeota*, and ILS200 and ILS300 represent the phylum *Bathyarchaeota*.

### Prediction of metabolic pathways in *Bathyarchaeota* assembled genomes.

The MAGs (ILS200 and ILS300) carried genes for carbon metabolism, nitrogen assimilation, oxidative phosphorylation, and degradation or assimilation of sugar, protein, and amino acids. In addition, the genomic potential of the assembled genomes corresponding to their metabolic pathways has been described.

### Carbon metabolism.

**FIG 3:**
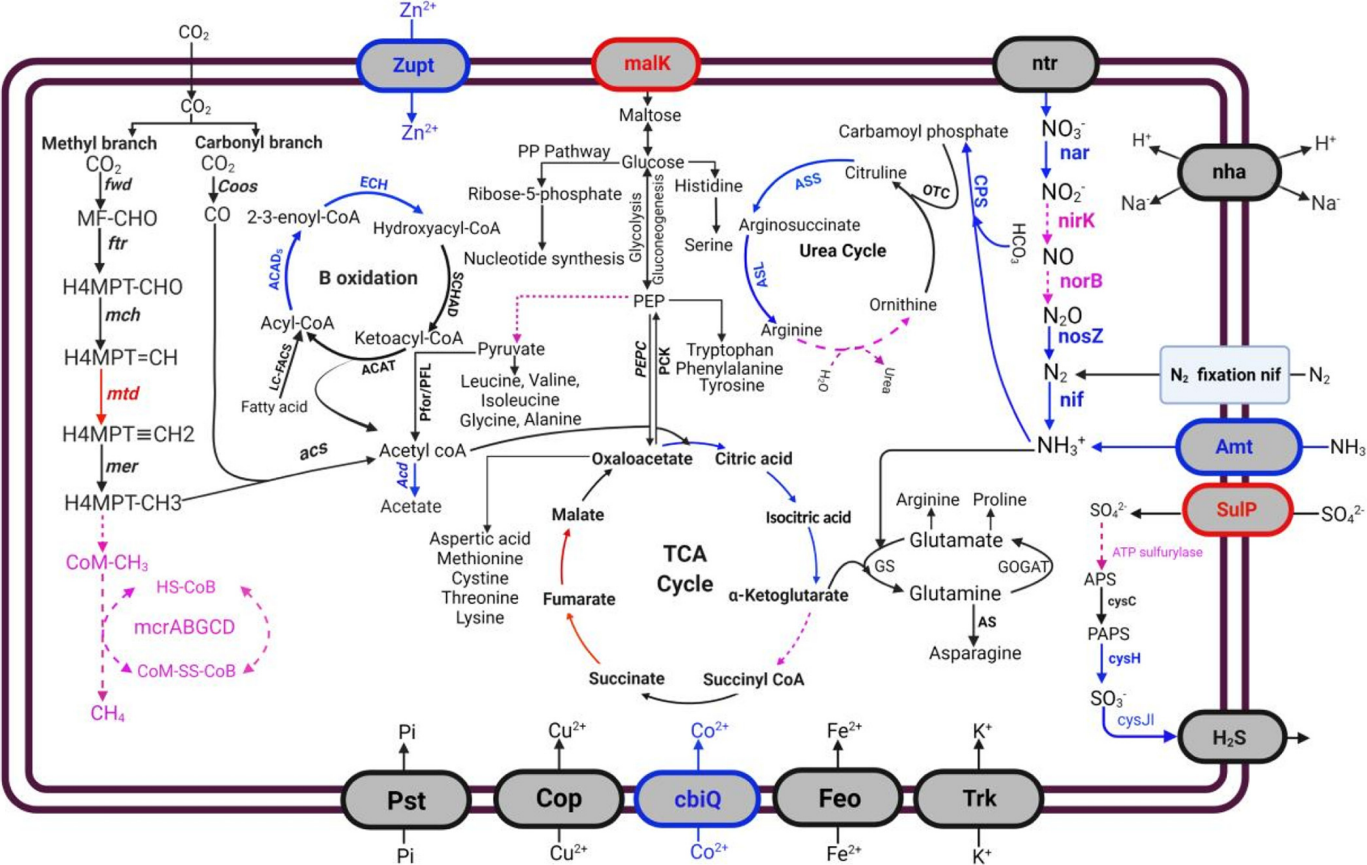
Key metabolic pathways in the MAGs of ILS200 and ILS300. ---, Genes absent in both the bins (purple color); genes found in both ILS200 and ILS300 (black), genes only absent in ILS200 (red), genes only absent in ILS300 (blue). Genes associated with the pathways highlighted in this figure are presented in Table S1 at https://figshare.com/s/d8c03fb25988b07c9479.

The *Bathyarchaeota* bins analyzed here encode genes in the Wood-Ljungdahl (WL) pathway, glycolysis, gluconeogenesis, the tricarboxylic acid (TCA) cycle, and the pentose phosphate pathway (Table S1 at https://figshare.com/s/d8c03fb25988b07c9479). In general, acetyl-CoA produced by sugar or protein degradation enters other metabolic pathways mainly through the bidirectional reductive acetyl-CoA or WL pathway. The WL pathway is crucial for archaea’s acetogenesis, methanogenesis, and carbon fixation (15, 17). The reconstructed genomes ILS200 and ILS300 identified the genes involved in the WL pathway. However, phosphotransacetylase (pta) and acetate kinase (ack) were not detected. It indicates the genomic potential for converting acetyl phosphate to acetyl-CoA by the enzyme phosphotransacetylase; eventually, acetate production by the catalytic activity of acetate kinase may not occur in these MAGs (6). Moreover, the genomic potential of ILS200 includes the acetyl-CoA synthetase gene (*acd*), which produces ATP and acetate, a trait commonly found in peptide-degrading archaeon *Pyrococcus* (20). Interestingly, both the bins carries genes for alcohol dehydrogenase and aldehyde ferredoxin oxidoreductase. These enzymes perhaps convert acetate to ethanol as an archaeal fermentation end product. Further, genes encoding the *mcr* complex were not detected in any of the bins, suggesting that these are incapable of producing methane. One of the reasons not to detect the coenzyme M reductase (MCR) complex in the draft genomes could be the result of reconstructing a fragmented genome from the metagenomic DNA. The fragmented assembly may predict a relatively higher number of short genes (fewer than 100 amino acids [aa]) than an isolated genome. Generally, the annotation pipeline missed short genes to assign the probable function (21). Instead, acetyl-CoA synthase (arCOG01340), carbon monoxide dehydrogenase/acetyl-CoA synthase complex (arCOG04408), and carbon monoxide methylating acetyl-CoA synthase complex beta subunit (arCOG04360) specific for acetate-forming archaea were present (22). It suggests the bins ILS200 and ILS300 that represent the phylum *Bathyarchaeota* solely depend on the WL pathway for synthesizing acetyl-CoA. Furthermore, the genomic potential of ILS200 and ILS300 showed the presence of tetrahydromethanopterin, which acts as a C_1_-carrier in nonmethanogenic archaea for carbon fixation (23) (Fig. 3). A conserved essential gene (*mcrA*) responsible for reducing the cofactor-bound methyl group to methane is absent. These MAGs might utilize sugars and amino acids for a heterotrophic lifestyle. Additionally, genes encoding the formate dehydrogenase were detected, suggesting the formation of acetyl-CoA through reductive acetyl-CoA pathways. The genomic potential of both the MAGs did not show the *mtr ABCDEFGH* operon, indicating that these are hydrogen-dependent methylotrophs. Thus, we predicted that the ILS200 and ILS300 genomes possess noncyclic carbonic fixation routes and produce acetyl-CoA from CO_2_ by the reductive acetyl-CoA pathway, finally utilized by the TCA cycle as a carbon or energy source.

Like other archaea, genes encoding phosphoenolpyruvate carboxylase (arCOG04435), phosphoenolpyruvate carboxykinase (arCOG06073), and phosphoenolpyruvate synthase (arCOG01111) were present. These enzymes could be involved in glucose metabolism (24). In addition, the genomic potential of both ILS200 and ILS300 showed genes encoding pyruvate formate-lyase, which indicates the formation of acetyl-CoA during anaerobic glycolysis (25). Surprisingly, pyruvate kinase was not found in any of the bins. Instead, a gene encoding l-alanine dehydrogenase was present, suggesting the possible role of this enzyme in the formation of pyruvate from alanine. Further, like ILS300, ILS200 carries ATP synthase, phosphoglycerate kinase, and several pyruvate ferredoxin oxidoreductase (porD) subunits, suggesting that ILS200 derives energy using both oxidative and substrate-level phosphorylation (26, 27). In addition, the genomic potential of both the MAGs showed genes encoding ribose 5-phosphate isomerase and orotidine 5′-phosphate decarboxylase. It suggests these genes are essential to the *de novo* biosynthesis of the nucleotides (28). Additionally, genes encoding cellulase/endoglucanase were also detected, indicating their ability to degrade polymeric carbohydrates.

### Nitrogen metabolism.

Ammonium is essential in microorganisms synthesizing nitrogen-containing metabolites such as amino acids. Like the hyperthermophile euryarchaeon Archaeoglobus fulgidus, genes encoding the ammonia transporter AmtB-like domain and nitrogen regulatory protein GlnK were detected in the genome of ILS200, indicating its ability to import NH_4_^+^ from the environment (29). In addition, genes encoding nitrogenase (arCOG00594), Mo-nitrogenase iron protein (arCOG00590), dinitrogenase iron-molybdenum cofactor biosynthesis (arCOG02734), and oxidoreductase/nitrogenase (arCOG00598) were detected in ILS200. It indicates that these enzymes play an active role in reducing N_2_ to ammonia (NH_3_), an essential step in nitrogen fixation (30, 31). Also detected were many genes involved in nitrogen metabolism, such as glutamine synthetase, glutamate synthase, asparagine synthetase A, and NADPH-dependent glutamate synthase subunit (GltB2, GltB3) (Fig. 3, Table S1 at https://figshare.com/s/d8c03fb25988b07c9479). These results suggested that both ILS200 and ILS300 carry out nitrogen metabolism by an assimilatory pathway, in contrast to their bathyarchaeotal homologs (32). Although nitrogen metabolism in archaea is less well known than that in bacteria, the availability of the complete genome sequences of a diverse group of archaea could help our future understanding of the physiology and biochemistry, including metabolic reactions involved in nitrogen compound utilization. Moreover, the genomic potential of ILS200 exhibited genes involved in the urea cycle, indicating its ability to eliminate the excess nitrogen or ammonia from the organism. Additionally, the genome predicted the enzymes in the biosynthesis of all 20 essential amino acids.

### Metal oxidation.

*Archaea* are capable of transforming the oxidation state of metals for bio-mineralization. Metal ions are required as a cofactor or used as the terminal electron acceptor in different biological processes. Several metal ion transportation genes, phosphate ABC transporter ATPase, phosphate ABC transporter permease, cobalt/nickel ABC transporter permease, cobalt transport protein, cobalt transport protein CbiM, cobalt ABC transporter inner membrane subunit CbiQ, copper-transporting P-type ATPase, Mn/Zn ABC transporter ATPase, magnesium-translocating P-type ATPase, K+ transporter Trk, Trk potassium uptake system protein, the ferrous iron transport protein B and iron ABC transporter permease, were identified in the genomes of ILS200 and ILS300. It indicated that these MAGs derived energy from reducing metals and metal ions, similar to other archaea (33) (Fig. 3).

### Other metabolic processes.

Metabolic predictions indicated that ILS200 showed flagellin genes for flagellar biosynthesis, which were absent in ILS300. Hence, both flagellated and nonflagellated “*Ca.* Bathyarchaeota” are present in the tropical hot springs. Loss of motility genes in ILS300 may be due to energy limitation or the changing oligotrophic environment of the hot spring ecosystem (34).

Fatty acid oxidation in archaea remains obscure. The genomic potential of the assembled genomes of ILS200 and ILS300 showed genes encoding acyl-CoA dehydrogenase, acetyl-CoA acetyltransferase, and enoyl-CoA hydratase of β-oxidation of fatty acids (Fig. 3). It suggests their ability to synthesize long-chain fatty acids to sustain themselves in an extreme environment (35).

ILS200 detected genes encoding beta-subunit of iron-sulfur flavoenzyme sulfide dehydrogenase (SudB). It suggests the ability to reduce elemental sulfur or polysulfide to hydrogen sulfide (36). These MAGs also encoded other structural genes and subunits of the assimilatory sulfate reduction pathway: sulfate permease (SulP), adenylylsulfate kinase (CysC), phosphoadenosine phosphosulfate reductase (CysH), sulfite reductase (CysJI) and thiosulfate sulfurtransferase rhodanese (Table S1 at https://figshare.com/s/d8c03fb25988b07c9479), and thioredoxin reductases (TrxR), suggesting that this enzyme is essential for regulating cellular redox balance and reducing the damage caused by reactive oxygen species generated via oxidative phosphorylation in the mitochondria (37). Additionally, thioredoxin reductases (TrxR) could be crucial in forming disulfide bridges to stabilize proteins, as found in hyperthermophilic organisms (38). Moreover, the genomic potential of both the MAGs encode enzymes involved in the benzoyl-CoA reductase complex, suggesting their ability to degrade aromatic hydrocarbon (39).

**Conclusion.** The presence of the WL pathway suggests that *Bathyarchaeota* bins (ILS200 and ILS300) could retain the capability to assimilate C_1_ compounds and generate acetate, ultimately contributing to the TCA cycle. However, a significant portion of the genes with a hypothetical nature is due to the incompatibility in the similarity search for novel functions. Nevertheless, most signature genes identified in archaeal genomes are ambiguous, or there are no homologs outside the archaea (40). Therefore, more genome sequences in the database may help to analyze the phylogenetically related but physiologically and functionally different archaea.

## MATERIALS AND METHODS

### Study site and sample description.

Samples collected from hot springs were geographically widely separated: Surajkund (24°09′01.9″N 85°38′45.2″E) main source (sample-1), Surajkund surrounding area (sample-2) located in the district Hazaribag, Jharkhand, India, and Bakreshwar (23°52′51.5″N 87°22′30.4″E) main source (sample-3) located in the district Birbhum, West Bengal, India. The temperature and pH of the three hot springs were recorded in the range of 67 to 83°C and 7.8 to 8.0, respectively. The highest temperature was recorded at Surajkund (main source) at 83°C, followed by Surajkund (surrounding area) at 72°C and Bakreshwar (67°C). The distance between Surajkund main source (sample-1) and Surajkund surrounding area (sample-2) was 6 m, and the distance between Surajkund and Bakreshwar was 221 kilometers (138 miles). Water temperature in the main source and surrounding areas was recorded using an Enviro-Safe thermometer (Sigma, USA). The pH was measured using a portable pH meter (Hanna Instrument, Sigma, USA). For DNA extraction, sediment samples (50 g) and water (50 mL) were collected in a sterile container from five locations in each spring. After collection, samples were pooled by mixing in equal proportions in sterile bottles.

### DNA extraction, sequencing, and data generation.

Purification of metagenomic DNA from pooled mixes of water and sediment samples were performed using a FastDNA spin kit for soil (BIO 101, California, USA) following the manufacturer’s instructions with minor modification. Briefly, silica beads were transferred from the Lysing MatrixE of the kit to a 15-mL sterile Falcon tube; 2.0 g of wet sediment, and 2 mL of lysis buffer (0.12 M sodium phosphate buffer, pH 8.0, 0.5% SDS) were added separately to the 20 sets of the tube for each sample for the extraction of DNA. Each tube was vortexed for 3 min and incubated at 65°C for 1 h. After lysis, the tubes were centrifuged at 2,300 × *g* for 20 min, and then the supernatant was transferred to a 2.0-mL sterile Eppendorf tube. This was then centrifuged at 14,000 × *g* for 10 min, and DNA in the supernatant was purified following the manufacturer’s instructions. DNA was eluted in 50 μL of DNA elution solution (DES) supplied with the kit. DNA extracts were pooled, and the concentration and purity were determined by measuring the absorbance ratios using a NanoDrop 8000 spectrophotometer (Thermo Scientific). The extracted DNA with a 260/280 ratio between 1.8 and 2.0 and a 260/230 ratio between 2.0 and 2.2 was considered pure. For high-throughput sequencing, a TG TruSeq Nano DNA HT library preparation kit (Illumina) was used to construct the paired-end sequencing library of metagenomic DNA. The metagenome sequencing was done using the Illumina HiSeq 4000 next-generation sequencing platform to produce paired-end sequence reads. The sequence quality was evaluated using the FastQC program (https://www.bioinformatics.babraham.ac.uk/projects/fastqc/) (41). *De novo* assembly of the sequences was performed using the MEGAHIT version 1.1.4 metagenome assembler (42).

A total of 7.5 GB, 7.2 GB, and 6.2 GB of sequence data from sample-1 (Surajkund, main source), sample-2 (Surajkund, surrounding area), and sample-3 (Bakreshwar) was obtained, respectively. Raw read sequence statistics such as read length and GC content of the processed reads were calculated using BBMap version 38.44 (43). The metagenomic sequences of each sample were filtered based on the following parameters: (i) quality filtration (phred quality, ≥Q15) and (ii) unique molecular identifier (UMI)-based elimination of duplicate data generated during Illumina sequencing; error correction was done using fastp tools version 0.20.0 (https://github.com/OpenGene/fastp) (44).

### Taxonomic and functional analysis of metagenome-assembled genomes.

Assembled contigs longer than 1 kb were binned to produce metagenome-assembled genomes (MAGs) using the MaxBin version 2.2.7 program (45). We examined the genome completeness by identifying the single-copy phylogenetic marker gene repertoire in the assembled genome (46). Further, we removed spurious genomes from the downstream analysis (47), and the quality and completeness of the genomes were estimated using CheckM version 1.0.7 with default parameters (48). The statistical elements, such as the number of scaffolds and the length of the assembled MAGs, were calculated using Perl script (https://github.com/tomdeman-bio/Sequence-scripts/blob/master/calc_N50_GC_genomesize.pl). The protein-coding genes (CDS) of the assembled genome (MAG) were identified by using the Prokka version 1.14.0 and the NCBI PGAP pipeline (release 2019-11-25.build4172), respectively (49, 50). We used the Barrnap version 0.9 program (https://github.com/tseemann/barrnap) with the parameter arch in the domain flag to predict the 16S rRNA gene sequence from the assembled archaeal genomic bin. Predicted protein-coding sequences were assigned to the archaeal clusters of orthologous genes (arCOGs) using the arCOG database (https://ftp.ncbi.nih.gov/pub/wolf/COGs/arCOG/) and the RPSBLAST algorithm implemented in the CDD2COG program (https://github.com/aleimba/bac-genomics-scripts/tree/master/cdd2cog) (51). The metabolic pathway was drawn using BioRender (https://help.biorender.com/en/articles/3619405-how-do-i-cite-biorender).

### Assessment of the ANI and the phylogenetic position of the MAGs.

ANI was calculated using the method described in Richter et al. (51) and implemented in the Python module PYANI version 0.1.2 (https://github.com/widdowquinn/pyani/releases/tag/v0.1.2). The 16S rRNA sequences were aligned using the SINA version 1.2.11 program (52) against the SILVA version 138.1 database (53), and the phylogenetic tree was generated using the ARB Parsimony (quick add marked) tool in the ARB software package (54). Further, the phylogenetic position of the three MAGs was determined using the 77 conserved marker proteins of archaea retrieved from 1,265 reference genomes from the database (www.ncbi.nlm.nih.gov/assembly/). First, the marker genes were extracted from the reference genomes using the AMPHORA2 pipeline (55), and the protein sequences were aligned using the MAFFT algorithm version 7.48 (56). Then, the aligned protein-coding sequences were concatenated, and the phylogenetic tree was built using IQ-Tree version 2.0.7 with the mixture model of LG + C60 + F + G and with ultrafast bootstrapping (-bb 1000, -alrt 1000) (57, 58). Finally, a phylogenetic tree was visualized with the ETE 3 tree viewer toolkit (59).

### Data availability.

The raw shotgun sequence reads are available in the NCBI-SRA database under the following accession numbers: SRR8368399 (Surajkund, main source), SRR8369092 (Surajkund, surrounding area), and SRR8369165 (Bakreshwar). All three metagenome-assembled genomes are available in NCBI GenBank under the following accession numbers: WUQR00000000 (ILS100), WUQU00000000 (ILS200), and WUQV00000000 (ILS300).
